# Efficacy of Emotionally Focused Therapy among Spanish-speaking couples: study protocol of a randomized clinical trial in Argentina, Costa Rica, Guatemala, Mexico, and Spain

**DOI:** 10.1186/s13063-022-06831-7

**Published:** 2022-10-22

**Authors:** Martiño Rodríguez-Gonzalez, Shayne Anderson, Alfonso Osorio, Marie-France Lafontaine, Paul S. Greenman, María Calatrava, Dania Andrade, Ragan Lybbert, Pilar Martínez-Diaz, Patrick Steffen, Jokin de Irala, Jonathan Sandberg

**Affiliations:** 1 Institute for Culture and Society (ICS), Universidad de Navarra (UNAV), Pamplona, Spain; 2 School of Education and Psychology, Universidad de Navarra (UNAV), Pamplona, Spain; 3 School of Family Life, Brigham Young University (BYU), UT Provo, USA; 4 School of Psychology, University of Ottawa (UO), Ottawa, ON Canada; 5 Département de psychoéducation et de psychologie, Université du Québec en Outaouais (UQO), Campus Gatineau, Gatineau, QC Canada; 6 Department of Psychology and UNINPSI, Comillas Pontifical University, Madrid, Spain; 7 College of Family, Home, and Social Sciences, Brigham Young University (BYU), Provo, UT USA; 8 Faculty of Medicine, Universidad de Navarra (UNAV), Pamplona, Spain

**Keywords:** Emotionally Focused Therapy, Couple therapy, Cultural adaptations, Spanish-speaking countries, Trials guidance

## Abstract

**Background:**

Couple relationship distress is common and associated with poor physical, psychological, and relational outcomes for both partners. Emotionally Focused Therapy for couples (EFT) is a short-term structured approach based on attachment theory that integrates a humanistic, experiential approach to restructuring emotional experience and a systemic structural approach to restructuring interactions. This model has been shown to be an effective treatment for couple distress. The supporting research, however, has only been conducted with English-speaking couples. Despite Spanish being the second-most spoken language and meaningful cultural differences between English- and Spanish-speaking countries, the efficacy of EFT has not been examined in this cultural context. This study will examine the efficacy of EFT in this particular context and advance the understanding of potential mechanisms of change.

**Methods:**

We will use a multicenter randomized wait-list controlled design to examine the efficacy of EFT in a Spanish-speaking sample of moderately distressed couples. One hundred forty individuals in 70 couples in Argentina, Costa Rica, Guatemala, Mexico, and Spain will be randomly assigned to receive 19–21 sessions of EFT or be placed on a waitlist. Outcomes on a range of relational and individual mental health variables will be assessed prior to random assignment, throughout treatment, and at the conclusion of treatment. Primary outcomes will include dyadic adjustment, couple satisfaction, and attachment. Secondary variables, such as loneliness, parenting, affective communication, and sexual satisfaction, will be included as potential mediators of the treatment effect. Couples in the treatment group will also be assessed at 3-, 6-, 12-, 18-, and 24-month follow-ups. Process variables such as the therapeutic alliance will also be assessed routinely in couples assigned to the treatment group. Couples in the waitlist will receive a psycho-educational program based on EFT after completing the study.

**Discussion:**

This study will be the first RCT of Emotionally Focused Therapy in a Spanish-speaking context. The results of the study will inform researchers interested in whether treatments developed and tested in the USA and Canada can be effective in differing cultural contexts. It may also point researchers and clinicians to areas where cultural adaptation is needed to improve efficacy.

**Trial registration:**

ClinicalTrials.gov NCT04277325. Registered on February 20, 2020

**Supplementary Information:**

The online version contains supplementary material available at 10.1186/s13063-022-06831-7.

## Administrative information

Note: the numbers in curly brackets in this protocol refer to SPIRIT checklist item numbers. The order of the items has been modified to group similar items (see http://www.equator-network.org/reporting-guidelines/spirit-2013-statement-defining-standard-protocol-items-for-clinical-trials/).

**Table:** 

Title {1}	Efficacy of Emotionally Focused Therapy among Spanish Speaking couples: Study protocol of a randomized clinical trial in Argentina, Costa Rica, Guatemala, Mexico, and Spain
Trial registration {2a and 2b}.	ClinicalTrials.gov Identifier NCT04277325
Protocol version {3}	Registered on February 20, 2020. Last Update on September 21, 2022
Funding {4}	This trial was funded by the University of Navarra, by Brigham Young University and by the International Centre for Excellence in Emotionally Focused Therapy
Author details {5a}	Martiño Rodríguez-González, Ph.D.^1^, Shayne Anderson, Ph.D.^2^, Alfonso Osorio, Ph.D. ^1^, Marie-France Lafontaine, Ph.D. ^3^, Paul Greenman, Ph.D.^4^, María Calatrava, Ph.D. ^1^, Dania Andrade, M.S. ^1^., Ragan Lybbert, M.S. ^2^, Pilar Martínez-Diaz, Ph.D. ^5^, Patrick Steffen, Ph.D. ^2^, Jokin de Irala, Ph.D.^1^, & Jonathan Sandberg, Ph.D. ^2^ ^1^ University of Navarra (UNAV) ^2^ Brigham Young University (BYU) ^3^ University of Ottawa (UO) ^4^ Université du Québec en Outaouais (UQO) ^5^ Comillas Pontifical University
Name and contact information for the trial sponsor {5b}	University of Navarra, Pamplona, Spain. Brigham Young University, Provo, Utah, USA
Role of sponsor {5c}	The sponsor, and not the funder, will have ultimate authority over all decisions.

## Introduction

### Background and rationale {6a}

Regardless of culture or country, the relationship individuals form with a spouse or partner is generally the longest and most influential relationship formed in adulthood [1]. Most adults want to be in a close couple relationship. Indeed, over 90% of those in Western cultures will marry by the age of 50. Yet, despite a desire for a close committed relationship, couple distress is common. We define couples’ distress as the predominance of reciprocal negative affect and lack of intimacy in a relationship that leads to both partners feeling dissatisfied and unable to meet each other’s needs. In the USA, between 20 and 31% of couples can be classified as distressed at any given time [2, 3]. In Spain, some studies suggest an even higher percentage of distressed couples among the general population [4]. Couple distress is associated with marital dissolution; depression; anxiety; substance abuse; poor performance at work; cardiovascular, immune, and endocrine system health; and mortality [5]. The resulting parental conflict can also have a direct impact on children’s academic performance, as well as on their social, emotional, and behavioral health [6]. It is not surprising, then, that relationship distress is one of the most cited reasons why individuals seek psychotherapy [7, 8]. For many couples, distress does not improve without intervention [9]. Fortunately, a significant body of research demonstrates that intervening with couples can significantly improve their relationships. A recent meta-analysis indicates that couple therapy is effective, leading to clinically significant improvements in communication, relationship satisfaction, and emotional intimacy [10].

While this is encouraging for couples in distress, it is important to note that this body of evidence comes from a very homogenous group—almost exclusively English-speaking couples residing in the USA or Canada. Despite calls from the American Psychological Association and others to establish the validity of treatments in different cultural contexts, the field of couple therapy has been slow to do so [11–13].

Spanish is the second-most commonly spoken native language in the world [14] and is the official language of 20 countries. Additionally, Latinos make up the largest ethnic minority in the USA [15]. Yet, despite these statistics, the field of couple therapy has done little to investigate the effectiveness of existing models within this cultural context or to identify cultural adaptations to address the needs of the Latino community [16]. Indeed, there are no randomized controlled trials of any couple therapy treatment provided in Spanish or in Spanish-speaking countries. The present study seeks to address this shortcoming by examining the efficacy of Emotionally Focused Therapy (EFT) for couples in Spanish-speaking countries.

EFT is a short-term (generally 20 or fewer sessions) treatment for relational distress that is grounded in attachment theory [17]. It is a humanistic intervention that combines an intrapsychic perspective with an interpersonal, systemic perspective to alleviate couple distress by helping partners increase their emotional accessibility, responsiveness, and engagement with their partner [17]. Treatment consists of three stages. During the first stage, the therapist helps the couple map and explore the negative pattern of interaction that is fueled by unmet attachment needs. During the second stage of treatment, the therapist restructures the interaction, helping each partner to be responsive to the vulnerable emotions shared by the other. In the final stage, gains made during treatment are consolidated [17].

EFT has been validated in Canada and the USA as an evidence-based treatment that has proven to be successful in helping distressed couples, including couples in which one partner is facing other health issues such as depression, PTSD, or terminal illness [18]. A recent meta-analysis found that EFT treatment produces a large effect (Hedge’s *g*= 2.09), with sustained improvement at follow-up [19]. Notably, EFT’s treatment effect is substantially larger than the medium effect size found in other meta-analyses that include all treatments for couple distress [10].

In addition to being among the most well-validated models of couple therapy, EFT appears to fit well within the cultural context of Latino families [16]. Sandberg and colleagues highlight the importance that Latinos place on social interactions that lead to emotional stability, harmony, and interdependence. They also point out that these elements fit very well with the primary foci of adult attachment theory which underlies EFT. Finally, these authors indicate that Latino couples place a high value on addressing emotions/emotionality in therapy, which, as the name indicates, is a core feature of EFT. No later than 2015, Spanish-speaking therapists from countries throughout the world began attending EFT trainings in Spanish. These trainings have been popular in Mexico, Panama, Costa Rica, Argentina, Spain, Ecuador, and Guatemala [16]. As EFT has already begun to take root within Spanish-speaking populations of the world, understanding the effectiveness of EFT among residents of Spanish-speaking countries grows increasingly important.

### Objectives {7}

The aim of this study is to determine whether EFT, compared to a wait-list control, leads to improved relational, physical, and psychological health among Spanish-speaking couples in Argentina, Costa Rica, Guatemala, Mexico, and Spain. A second research goal is to study the potential mechanisms of change in order to understand how change occurs in EFT.

### Trial design {8}

This study uses a multicenter randomized non-inferiority wait-list controlled design. We anticipate recruiting 70 couples (140 individuals) into the study who will be allocated on a 1:1 ratio to the treatment group which will receive 19–21 sessions of emotionally focused couple therapy, or to the wait-list control group. Group allocation will not be blinded.

## Methods: participants, interventions, and outcomes

### Study setting {9}

The interventions of this study for the treatment group will take place in community counseling or mental health offices/clinics where the participating therapists are already practicing within each participating country—Argentina, Costa Rica, Guatemala, Mexico, and Spain. The detailed list of study sites can be obtained in the project registry at clinicaltrials.gov [20].

### Eligibility criteria {10}

Inclusion and exclusion criteria for participants are detailed in Table 1 and reflect the eligibility criteria used in other studies about EFT conducted in English-speaking countries [18, 21].

**Table 1:** Participant eligibility

**Inclusion criteria**	
1. Couples who have been in an exclusive relationship and living together for at least 1 year	
2. Both members of the couple must be over 25 years old (there is no upper limit for participants’ age)	
3. Both members are willing to participate in all aspects of the study, including completing questionnaires, being videotaped in therapy, attending therapy, and participating in the follow-up after treatment has been completed	
4. Both members of the couple are native Spanish speakers and have lived in one of the included countries (Argentina, Costa Rica, Guatemala, Mexico, or Spain) for a minimum of 5 years prior to participating	
5. The average score of the couple’s dyadic adjustment, measured by the Dyadic Adjustment Scale (DAS, see the “Outcomes {12}” section below) falls between mildly (80) to moderately (100) distressed	
**Exclusion criteria**	
Either partner:	
1. Is receiving current treatment through psychotherapy at the time of recruitment or anticipates doing so outside of the proposed study within the next 6 months	
2. Has been previously diagnosed with any psychotic, somatoform, or dissociative disorder	
3. Is taking medication known to treat psychosis, somatoform, psychotic or dissociative disorders or is taking a psychotropic medication	
4. Is misusing drugs or alcohol, defined as frequent (more than once a week) and maintained (for more than a year) use that has led to a work or personal problem	
5. Has a diagnosis of a neurodevelopmental, neurocognitive, personality, or paraphilic disorder	
6. Reports having been arrested or in prison in the past 3 months	
7. Reports losing her/his employment due to alcohol or drug-related problems	
8. Reports an episode of sexual assault (as victim or perpetrator) in their life during the last 2 years	
9. Reports current physical or sexual violence in their relationship	
10. Is currently involved in an affair which she/he is unwilling to disclose to her/his partner and/or terminate	
11. Has or anticipates circumstances which will make attending therapy sessions unlikely, such as major surgery expected in the next 3 months, or moving to a new area in the near future, etc.	
12. Is a psychotherapist in active clinical practice	
13. Has a direct knowledge of EFT because they are currently receiving training or have been trained in EFT	

### Who will take informed consent? {26a}

During the first interview with the therapist, the therapist will ask for written consent from each partner separately. One copy of each partners’ informed consent form will be kept for study documentation and another copy will be given to each individual for their records.

### Additional consent provisions for collection and use of participant data and biological specimens {26b}

Participants have consented to allow responses to all questionnaires as well as video recordings of their sessions to be used for the current outcome study as well as in future analyses of the change process. No additional consent provisions are anticipated.

## Interventions

### Explanation for the choice of comparators {6b}

A wait-list control group was chosen for this study because there are no previous randomized controlled trials of any intervention for couple distress conducted in Spanish outside the USA. Thus, one of the primary goals of this study is to identify whether EFT for couples is an effective treatment outside of the USA and Canada, where it was developed. Comparison against no treatment is a necessary preliminary step that replicates the development of EFT in English clinical trials.

Since all couples in this study report distress in their relationship, careful consideration was given to minimize the potential impact of not receiving treatment in the control group couples. First, couples with severe distress are excluded from the study and recommended to pursue therapy. Second, couples that meet the inclusion criteria of mild to moderate distress are provided a psycho-educational program called “Hold Me Tight” after the post-test. This program is based on the same principles as EFT and was designed to improve couple relationships (see the section “Participant timeline {13}”). Finally, participants are informed about their right to withdraw from the study if immediate treatment is needed. In two previous studies of EFT that used similar inclusion criteria and a no-treatment control, no control group couples withdrew from the study [22, 23].

### Intervention description {11a}

Couples assigned to the treatment group will receive between 19 and 21 sessions of EFT. These sessions will take place at one of the therapy sites involved in the study and will be conducted by a participating therapist selected by the research team. Therapy sessions for the treatment group will preferably take place weekly, but when this is not possible, every other week. Both partners will be present at the majority of sessions, except for two individual meetings (one with each partner) which will happen between sessions 2 and 4. Sessions will last approximately 75 (70–80) min. Participating therapists will provide therapy with the maximum possible fidelity to the rules and instructions of the EFT model.

EFT will be administered by couple therapists who will either be therapists certified by the International Centre for Excellence in Emotionally Focused Therapy (ICEEFT) or candidates for certification completing the supervised practice requirement. In addition, all therapists will be native Spanish speakers, conducting their clinical practice in a Spanish-speaking country, with an active license to practice psychotherapy in their country of residence (Argentina, Costa Rica, Guatemala, Mexico, or Spain).

All therapists will be requested to offer their services on a voluntary basis. Therapy will be conducted at their clinic, and each of the sessions will be video recorded. However, therapists will be reimbursed for the time spent in duties linked with research processes (e.g., sending questionnaires or video recordings, checking processes with the research team, etc.).

To participate in this study, therapists will be required to receive weekly or biweekly group and/or individual supervision with an EFT-certified supervisor. This supervision will be carried out in Spanish.

### Criteria for discontinuing or modifying allocated interventions {11b}

After the random allocation, and before the start of the treatment, the team will endeavor to book appointments that fit couples’ and therapists’ schedules. If it is impossible to find a compatible appointment time, a different therapist will be assigned to the participating couple, if available.

During the intervention, if new circumstances arise that might necessitate the exclusion of the couple (e.g., meeting an exclusion criterion, or no longer meeting an inclusion criterion), the therapist will provisionally interrupt the therapy. Then, together with the PI, they will decide whether the criteria are met and whether the couple may continue in therapy or must leave the study.

All these changes will be documented and considered in the statistical analyses of the data.

### Strategies to improve adherence to interventions {11c}

In order to ensure the therapists in this study are implementing EFT faithfully, two different procedures will be carried out. First, therapists will receive weekly or biweekly supervision from EFT-certified supervisors. During these meetings, the supervisor will review video recordings of therapy sessions to determine if EFT is being conducted according to the model and to provide corrective feedback. Second, fidelity will be assessed by two independent judges using an EFT intervention checklist [24]. Judges will randomly rate five sessions of each therapist’s taped sessions to ensure that at least 80% of the therapists’ interventions can be coded as adherent to the model.

### Relevant concomitant care permitted or prohibited during the trial {11d}

Concomitant care will be prohibited. Couples in the control group will not be allowed to receive any kind of therapeutic couple intervention during the trial. Couples in the treatment group will not be allowed to receive any couple intervention outside the intervention received in the trial. The failure to comply with this condition will result in exclusion from the study.

### Provisions for post-trial care {30}

Therapy involves answering questions about thoughts and emotions, as does the completion of study questionnaires. Participants might experience some mild discomfort in responding to them, but no more so than if they were to remember a sad event in their lives. If for any reason this were to happen and the discomfort were to become difficult to manage, participants who are receiving EFT will be encouraged to discuss this difficulty with their therapist. Participants in the control group will be given the contact information for the principal investigator (PI), who is a registered psychologist, should they wish to address any discomfort that might arise. For these reasons, harm resulting from trial participation is not expected, and compensation or post-trial care is not planned.

### Outcomes {12}

Most of the outcome measures used in this trial have been adapted to Spanish-speaking populations, with successful validation studies. In some other cases, adaptation has been made, but validation is still pending. In each outcome, scores will be calculated according to the authors’ instructions. For the main analysis, the metric will be the change from baseline to the end of the intervention, and this change will be compared between both groups. Additional analyses will consider other time points (see Table 2).

**Table 2:** Study schedule of enrolment, interventions, and assessments

	Study period																											
	Enrolment	Pre-allocation	Allocation	Post-allocation																				Follow-up				
Timepoint		***t***_***0 baseline***_		***t***_***1***_	***t***_***2***_	***t***_***3***_	***t***_***4***_	***t***_***5***_	***t***_***6***_	***t***_***7***_	***t***_***8***_	***t***_***9***_	***t***_***10***_	***t***_***11***_	***t***_***12***_	***t***_***13***_	***t***_***14***_	***t***_***15***_	***t***_***16***_	***t***_***17***_	***t***_***18***_	***t***_***19***_	***t***_***20***_	***3m***	***6m***	***12m***	***18m***	***24m***
**Recruitment**																												
Eligibility screen	A	A																										
Informed consent		A																										
DAS-32	A	A											C	T									A			T		
Allocation			A																									
**Interventions**																												
EFT				T	T	T	T	T	T	T	T	T	T	T	T	T	T	T	T	T	T	T	T					
**Assessments**																												
Demographics 1	A																											
Demographics 2		A																										
DAS-4				T	A	T	C	T		A		T				A		T		T	C	T		T	T		T	T
CSI-16		A				T	C			A			C	T				T	C				A	T	T		T	T
ECR-36		A					A		T	C			A				A		A		A		A	T	T	T	T	T
Health-4		A					A			T	C				A					A			A	T	T	T	T	T
PHQ-15		A									T	C						A					A	T	T		T	
DASS-21		A		A							A			T		C			T	C			A	T	T	T		T
SD-13		A						A							A					A			A	T	T	T		T
UCLA LS-R-8		A			T	C		A				A		T	C			C	T				A	T	T	T	T	
RFQ-8		A			T	C		A			A			T	C			A					A	T	T	T		T
AP15		A			C	T			C	T							A						A		T		T	
Sleep-8		A			C	T			A							A					A			T	T	T		T
CORE-10		A		T			T	C			T	C			T	C			A			T	A		T		T	
NEO-N12		A										T										T		T			T	
DSI-26		A			T	C			A				C			T	C				A		A		T	T		T
RELATES-55		A											T	C									A		T		T	
SLEs-15		A							C	T							A						A	T		T		T
BARE12					A			T		C		T				A			C	T			A			T		T
WAI-Co16^a^				T		T		T		T		T		T		T		T		T		T						
ABAQ12^a^							T				T				T				T			T						
IAS-C4^a^				T	T		T		T		T		T		T		T		T		T		T					
PSRQ^a^				T	T	T	T	T	T	T	T	T	T	T	T	T	T	T	T	T	T	T	T					

*T* treatment group, *C* control group, *A* all participants

^a^WAI-Co16, ABAQ12, IAS-C4, and PSRQ are therapy-related measures and will not be used in the control group

### Primary outcomes: dyadic adjustment, couple satisfaction, and attachment

- Dyadic adjustment will be assessed using two versions of the Dyadic Adjustment Scale (DAS-32 and DAS-4)
  The Dyadic Adjustment Scale (DAS) [25] is a 32-item measure of romantic relationship adjustment. Partners are asked to rate the occurrence of both relationship disagreements and positive relationship exchanges on a 5- or 6-point Likert scale. Higher scores on this measure are indicative of better relationship adjustment (DAS-32) or higher relationship satisfaction (DAS-4). The full version will be used at the pre-test, midpoint, and post-test for all participants. In order to minimize participant burden, a short validated version (DAS-4) [26] will also be used to assess relationship satisfaction at all other measurement periods throughout the study.
- Couple Satisfaction Inventory (CSI-16)
  The CSI-16 [27] is a 16-item measure of relationship satisfaction. One global item uses a 7-point scale, whereas the other 15 items use a variety of response anchors, all with 6-point scales. Higher scores indicate higher levels of relationship satisfaction.
- Experiences in the Close Relationships Questionnaire (ECR-36)
  Romantic attachment was assessed using the ECR [28], which measures romantic attachment using 36 items along two subscales: attachment anxiety and attachment avoidance. Participants evaluate the extent to which they identify with each item using a 7-point Likert scale from 1 (strongly disagree) to 7 (strongly agree). Once respective items are reversed, the score of each subscale corresponds to the mean of its items. Higher scores indicate greater levels of attachment anxiety and attachment avoidance. Items have been edited to refer to the current couple relationship.

#### Secondary outcomes (control, predictive capacity, and change)

We will also conduct an analysis of secondary variables in order to test their influence as predictors of the main outcomes, as potential relevant variables in the process of change, and as control variables.

- General Health Questions (Health-4)
  In this measure, respondents are asked to rate their physical health. Higher scores on this measure represent better health. Participants respond to these questions about general health based on a 5-point Likert scale ranging from 1 (poor) to 5 (excellent).
- Patient Health Questionnaire (PHQ-15)
  The PHQ-15 is a somatic symptom subscale which stems from the original full PHQ. It asks about 13 somatic and 2 psychological (fatigue, sleep problems) symptoms. Each item is scored from 0 (not bothered at all) to 2 (bothered a lot). Items consist of symptoms such as “stomach pain” and “headaches” [29].
- Depression Anxiety Stress Scale (DASS-21)
  The DASS-21 consists of 21 negative emotional symptoms statements/questions. Respondents rate to what extent over the past week they have experienced each symptom on a 4-point scale of severity or frequency. The DASS-21 has subscales for depression, anxiety, and stress. Items on the depression subscale consist of statements like “I felt that life was meaningless” and “I felt down-hearted and blue.” Items on the stress subscale consist of statements like “I felt I was rather touchy” and “I found it difficult to relax.” Items on the anxiety subscale consist of statements like “I felt I was close to panic” and “I felt scared without any good reason” [30].
- Sexual Dissatisfaction subscale of the Marital Satisfaction Inventory (SD-13)
  The Revised Marital Satisfaction Inventory measures the type and severity of relationship distress in multiple areas of marital interaction. Respondents choose between a true and false response to each item. The Sexual Dissatisfaction (SD-13) subscale assesses the level of dissatisfaction through statements regarding the frequency and quality of the couple’s sexual activity. Items consist of statements such as “My spouse sometimes shows too little enthusiasm for sex” and “My spouse has too little regard sometimes for my sexual satisfaction” [31].
- UCLA Loneliness Scale Revised - Short version (UCLA LS-R-8)
  The revised UCLA loneliness scale (UCLA LS-R-8) is an 8-item questionnaire designed to measure and detect variations in loneliness. Each item is ranked on a 4-point Likert scale ranging from “Never” to “Often.” Items consist of statements like “I feel isolated from others” and “I lack companionship” [32].
- Reflective Functioning Questionnaire (RFQ-8)
  The RFQ seeks to measure the capacity to understand one’s own and others’ feelings, goals, and attitudes. It has an uncertainty subscale and a certainty subscale. This short version (RFQ-8) contains 8 items which are scored on a 7-point Likert scale ranging from “strongly disagree” to “strongly agree.” Items consist of statements such as “People’s thoughts are a mystery to me” and “I always know what I feel” [33].
- Authoritative Parenting subscale (RELATE)-(AP-15)
  The Authoritative Parenting subscale comes from the Parenting Style and Dimensions Questionnaire Short form (PSDQ). The PSDQ is used to measure parenting styles. The authoritative parenting subscale from the short version of the PSDQ is composed of 15 items [34, 35]. Items include phrases such as “I am responsive to our child’s feelings or needs” and “I allow our child to give input into family rules.” Responses are scored on a 5-point Likert scale ranging from “Never” to “Always” [35]. This instrument will be only administered to couples with children.
- Sleep Quality (Sleep-8)
  The PROMIS sleep disturbance short form (Sleep-8) questionnaire has 8 items. Respondents are asked to assess their sleep quality over the past 7 days by responding to statements like “I had trouble sleeping” and “My sleep was refreshing.” Responses are scored on a 5-point Likert scale ranging from “Not at all” to “Very Much” [36].
- CORE Outcome Measure short form (CORE-10)
  The CORE-OM is a 34-item client self-report questionnaire designed to be administered before and after therapy. The CORE-10 is a brief outcome measure comprising 10 items drawn from the CORE-OM. The CORE-OM has been widely adopted in the evaluation of counseling and the psychological therapies in the UK. The CORE-10 taps global distress and is, therefore, suitable for use as an initial quick screening tool and also as an outcome measure.
- NEO Five-Factor Inventory (NEO-N12)
  The revised NEO Five-Factor Inventory is an inventory seeking to measure the 5 basic traits of personality as described by the “Big 5” personality traits theory. The “Big 5” traits include openness, conscientiousness, extraversion, agreeableness, and neuroticism [37]. The neuroticism subscale selected comes from the Five Factor Inventory [38], intended to measure an individual’s level of neuroticism. It has an internal reliability of .83. Items are scored on a 5-point Likert scale ranging from “Strongly Disagree” to “Strongly Agree” [39]. Higher scores indicate higher levels of neuroticism (emotional instability). Items include statements like “I often feel tense and jittery” and “sometimes I feel completely worthless.” This measure would provide data on the mental health of participants within the study. The importance of mental health and how it relates to this study has been outlined previously.
- Spanish Differentiation of Self Inventory (S-DSI-26)
  The Spanish Differentiation of Self Inventory is a 26-item questionnaire which measures the degree to which one is able to balance emotional and intellectual functioning as well as balance autonomy with intimacy in relationships. It has an overall internal reliability of .88. It has two subscales, one which measures emotional reactivity (internal reliability of .88) and the other emotional cutoff (internal reliability .79). Responses are scored on a 6-point Likert scale ranging from “Not at all true for me” to “Very true for me.” Items include statements like “I wish that I were not so emotional” and “I am overly sensitive to criticism” [40, 41]. This measure would provide data which would allow researchers to assess the relationship between important personal emotional and relational qualities which may contribute to (or detract from) relationship quality. Additionally, the impact of EFT on these variables could be studied.
- RELATE measure (RELATES-55)
  In the current study, we are using 12 subscales from the RELATE measure. In most cases, we will assess both the actor (self) and partner perspectives for each item. The RELATE measure has undergone rigorous psychometric evaluation over a nearly 30-year period [42, 43].
- Stressful Life Events (SLEs-15)
  Couples’ experience of stressful life events is assessed using 14 items related with major stressful life events and one final question about the perceived impact of these events on the couple relationship. Moreover, participants are asked to report the kind of impact the event could have in their relationship. Examples of events are “death of a child” or “change in residence.” Economic stress is also measured with two items. All items are reverse coded so that higher scores indicate higher levels of stress experienced (i.e., more stressors experienced). It is important to control the effect of the major stressful life events could have in the therapy process. The SLES-15 was developed for this study.
- The Brief Accessibility, Responsiveness, and Engagement Scale (BARE-12)
  The BARE is an instrument which measures an individual’s perception of their own and their partner’s attachment behaviors. It has 12 items and measures attachment behaviors through 3 subscales: accessibility, responsiveness, and engagement (for both self and partner), all of which are related to secure attachment. Items are scored on a 5-point Likert scale ranging from “Never True” to “Always True.” Items consist of statements such as “I am rarely available to my partner.” and “I am confident my partner reaches out to me.” [43].

#### Process variables: understanding of the change process in EFT

We will also include analyses that will allow us to study the process of change, to better understand session-to-session micro-level shifts in couple dynamics and intrapersonal wellbeing. In order to achieve this, we will gather (only in the intervention group) data regarding variables such as the therapeutic alliance, as well as clients’ perception of partner accessibility, responsiveness, and engagement along the course of therapy. These process variables will also be linked to outcomes.

- Working Alliance Inventory for Couples Short Form (WAI-Co16)
  The Working Alliance Inventory for Couples Short Form (WAI-CO) contains 24 questions. It was designed to measure the therapeutic alliance in couple therapy and how well couples and therapists align on 3 subscales: goals, tasks, and bond. Responses are scored on a 7-point Likert scale ranging from “Never” to “Always.” The WAI-CO has good (.95) internal reliability [44]. The short version of the working alliance inventory has good convergent validity [45]. Items include statements like “My partner and the therapist trust one another” and “The therapist and I agree about how best to use the time in therapy.” In the current study, we are going to use only sections 1 and 2 of the questionnaire (16 items). Therapeutic alliance (working alliance) has been established as an important predictor of therapeutic outcome. This inventory would allow researchers to assess for the impact of EFT on the therapeutic alliance and its subscales amongst therapy clients living in Spanish-speaking countries. The relationship between therapeutic alliance and therapeutic outcomes among the same population could also be studied.
- The Attachment Based Alliance Questionnaire (ABAQ12)
  The Attachment Based Alliance Questionnaire (ABAQ) uses attachment theory as the theoretical footing for the assessment of the therapeutic alliance. It is a 12-item questionnaire scored on a 7-point Likert scale ranging from “Completely agree” to “Completely disagree.” It contains 2 subscales, an attachment anxiety and an attachment avoidance subscale. The ABAQ has good internal reliability (.88). It has been shown to have good convergent and discriminant validity. Higher scores indicate a stronger therapeutic alliance. Items include statements like “My therapist wants to know too much about me” and “I worry about my therapist abandoning me.” [46]. This questionnaire will allow further insight into the therapeutic alliance when working with therapy clients living in Spanish-speaking countries by allowing for an assessment of the relationship between attachment styles and their alliance with their therapist. As EFT is an attachment-based theory, this measure will add important data as to how EFT affects the therapeutic alliance among therapy clients in Spanish-speaking countries.
- Alliance Scale-Couple version (IAS-C4)
  The Intersession Alliance Scale-Couple version (IAS-C) is a four-item visual analog scale designed to measure the bonds, goals, and tasks, within system, and safety within the system dimensions of the therapeutic alliance. Each dimension is measured on a scale that ranges from 0 to 100 with an average item score used as the total alliance score. The IAS-C has demonstrated excellent criterion, content, and predictive validity as well as strong reliability [47].
- Post Session Resolution Questionnaire (PSRQ)
  The Post Session Resolution Questionnaire (PSRQ) is an adapted version of [48] Therapy Session Report Questionnaire. The PSRQ asks partners to rate how well the session topics related to their therapeutic goals, and how much they thought the session moved them towards resolution of their problems. The PSRQ contains four items, three of which are rated on a 5-Likert scale, and one of which is rated on a 7-point Likert scale. This measure only has face validity and has been used in previous studies [49, 50] to identify the best and worse sessions for the use of psychotherapy process measures such as the SASB [51] and the ES [52]. Three of the questions are summed together for a PSRQ change score, where higher scores are indicative of higher perceived levels of change. This questionnaire would serve as an additional assessment of the therapeutic alliance and how therapy clients in Spanish-speaking countries felt that EFT was able to address their presenting problem in the therapy context.

### Participant timeline {13}

**Fig. 1:**
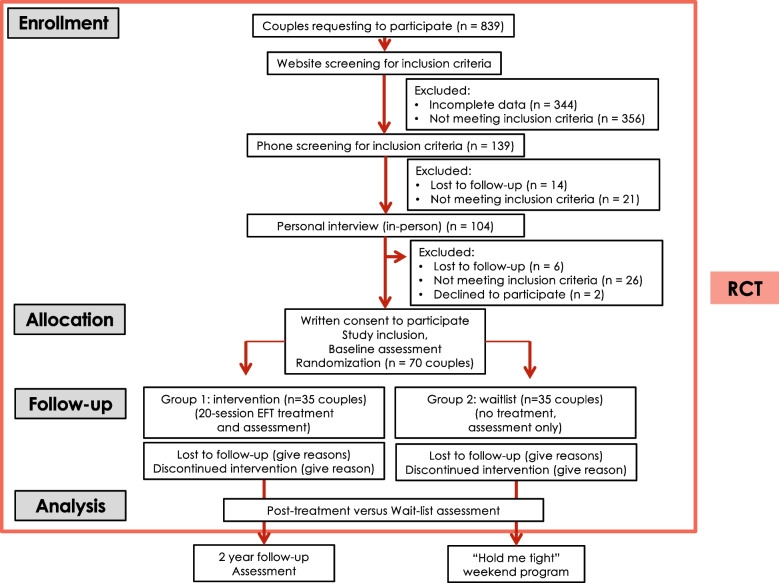
Figure flowchart

Couples in the treatment group will receive 19–21 sessions of EFT over 5–6 months. Couples in the control group will receive online questionnaires with a similar periodicity. After treatment, couples in the treatment group will be invited to participate in a follow-up assessment at months 3, 6, 12, 18, and 24, and couples in the control group will have the opportunity to participate in a psycho-educational weekend program called “Hold Me Tight” (HMT) (Fig. 1).

Details about the timeline and assessments are reported in Table 2.

### Sample size {14}

A sample size of 70 couples (35 per group) was determined by conducting power estimates for multilevel modeling (MLM) analysis which will be used to address the primary outcomes of this study.

The use of MLM as the method of analysis is relatively new to couple research, and many of the published studies that were conducted tested two-level models [53–56]. For the sample size estimation in the current study, we considered the simulation study by Mass and Hox [57] and previous studies that have used two-level MLM models with couples. Mass and Hox [57] suggested that, when testing a two-level model with a medium effect size with *N*= 30, the 0.05 alpha level for the slope is overestimated at 0.088. However, Stevens [58] highlights that averaged parameter estimates that occur at the third level of a model are considered more reliable, as they are derived from couples’ averaged scores rather than individual scores.

This study will use a three-level model. We anticipate that the effect size will be medium to large based on previous effect sizes in EFT studies as well as Dunn and Schwebel’s [57] average effect size of all couple therapy interventions .90 on global measures of relationship satisfaction. To ensure power at .80, the aim of the current study is to employ a sample of 70 couples equally allocated between the treatment and control groups. We also expect to have minimal attrition across both treatment and control conditions during the intervention phase of the study. This is consistent with previous clinical trials of EFT [22, 23].

### Recruitment {15}

Participants for this study will be recruited through a number of potential outlets including, but not limited to, media advertisements, posters at local community agencies, and other professional and social networks depending on the specific location of the therapists involved in the clinical trial. The recruiting process may also include an advertisement for the study on other social media outlets or internet sites, i.e., a video shared through YouTube and social networks (Instagram and Facebook), as appropriate by location.

All respondents to these recruitment methods will be screened for eligibility through an online questionnaire and through a standardized telephone screening procedure. Selected respondents will meet a participating therapist who will confirm inclusion and exclusion criteria during a face-to-face interview with each partner, explain the study, seek confirmation that the couple understands what participating in the study will entail, and ask the respondents if they still wish to participate in the study. If so, the respondents will be asked to sign informed consent forms.

## Assignment of interventions: allocation

### Sequence generation {16a}

Allocation will occur via block randomization by location.

A member of the team (the sequence generator) will list all possible sequences of *X* elements (*X* being the block size) in which each of the two elements (A and B) appears *X*/2 times. The sequence generator will then order the blocks randomly, from one to *N* (*N* being the number of possible blocks).

### Concealment mechanism {16b}

Couples recruited within each location will be assigned by the research team a unique ID, consisting of one or more letters to designate the location (e.g., M for Madrid) and a sequentially numbered value in order of entry into the study. Therefore, for Madrid, couples will be M1, M2…

Allocation to groups will be determined by the group allocator, a researcher who is not otherwise associated with the study. The group allocator will be only given the anonymous list of couple IDs and the list of possible sequences, randomly ordered. The group allocator will not have access to the key matching IDs to couples.

The sequence generator and the group allocator will be different people to ensure the integrity of concealment and sequence generation.

### Implementation {16c}

The group allocator will receive written instructions to run the allocation. In each block of participants (in each location), the group allocator will assign a letter (A or B) to each couple through the following steps:

1. The group allocator will consult the list of sequences provided in the instructions packet to determine the possible sequences for assignment. In this list, each sequence has a number assigned.
2. Using a computer program (Microsoft Excel), the group allocator will generate a randomized number from the range of provided sequences for the block (a number between 1 and *N*). This random number will indicate which sequence the group allocator should use.
3. Using the same program, the group allocator will generate a random number (ranging from 1 to 2). Number 1 will represent A, and number 2 will represent B. The number generated by the online program will be the treatment group. For example, if the program returns number 1, couples with an assigned A will compose the treatment group.
4. The group allocator will record each step in a template document, will sign this record, and will deliver it to the PI.

## Assignment of interventions: blinding

### Who will be blinded {17a}

Given the nature of the intervention, neither therapists nor participants can be blinded after assignment to interventions. Still, data analysts will be blinded by creating syntaxes before adding the treatment condition variable to the data set.

### Procedure for unblinding if needed {17b}

The design is not blind, so unblinding will not occur.

## Data collection and management

### Plans for assessment and collection of outcomes {18a}

The assessment plan is detailed in Table 2. Participants will have the option to complete questionnaires either from home, through the Qualtrics online assessment platform, or from the therapist’s office using a paper-and-pencil version. However, to minimize missing data, data from the pre-test and post-test will be collected in-person by the therapist. Partners will be encouraged to complete the questionnaires independently to ensure confidentiality. Participants in both conditions will receive the same measures with the exception of those that are unique to the process of therapy (e.g., therapeutic alliance or post-therapy questionnaire). Pre-test and post-test assessments will take approximately 30–60 min, study period assessment will take around 10–20 min, and follow-up assessments for the treatment group will take approximately 30–45 min to complete.

### Plans to promote participant retention and complete follow-up {18b}

Several strategies will be used to promote retention and minimize missing data. Members of the research team will send reminders about therapy sessions to couples in the treatment group. Automated notifications are sent to the research team when a therapy session has been completed and when online assessments have been completed to facilitate tracking of data for both treatment and control groups. A member of the research team will also send reminders to complete questionnaires. Furthermore, compensation will be provided for time couples spend in research protocols.

### Data management {19}

There will be different data collection strategies:

- Participants and therapists responding to questionnaires online. These data will be automatically transferred to the database.
- Participants responding to paper-pencil questionnaires at the therapist’s setting. Therapists will send the completed questionnaires to the research team, who will manually enter these data into the database.

Data management and primary analyses will take place at both the University of Navarra (UNAV) in Pamplona, Spain, and at Brigham Young University (BYU) in Provo, UT, in the USA.

### Confidentiality {27}

Strict data storage and protection of confidentiality will be observed. Physical data (hard copies of completed questionnaires, consent forms, therapists’ notes, video recordings of therapy sessions, etc.) will all be kept in a locked filing cabinet in each therapist’s clinic. Five years after the end of the study, they will be destroyed. Access to these materials will be limited to the corresponding therapist and the PI. Digital data will be temporarily saved by each therapist strictly on devices with secure password protection which will be stored at their clinic. These data will be transferred monthly through a secure file transfer protocol (SFTP). All files will be encrypted before being uploaded, and they will be password protected. This password will be conveyed to the principal investigator’s laboratory (UNAV) through a separate electronic medium. Once accurate receipt of the digital files has been confirmed, the therapist who sent the files will delete all electronic/digital information.

Electronic information generated during this study will be stored in two separate locations: on a local hard disk, which will be stored in a locked filing cabinet in the PI’s office, and on an online server with advanced security features which meet or exceed the European Union’s General Regulation standards. This electronic information will be shared with members of the research team at BYU through SFTP. Research team members at BYU will temporarily store the electronic files on a secure server. All files will be protected through secure passwords.

All questionnaires and materials containing personal information will be deidentified using unique couple identification numbers and partner designations. No name or personal information linked to the subjects’ identity will be placed on the questionnaires. A list linking couple identification numbers and names will be stored on a password-protected computer in the PI’s lab at the University of Navarra. The PI and two members of the research team will be the only individuals who have access to this list.

### Plans for collection, laboratory evaluation, and storage of biological specimens for genetic or molecular analysis in this trial/future use {33}

Not applicable.

## Statistical methods

### Statistical methods for primary and secondary outcomes {20a}

Three-level multilevel linear models (MLM), with Bayesian estimation, will be used to test our primary and secondary outcomes. MLM is a growth-modeling analysis (GMA) that is frequently used in clinical trials of psychological interventions with repeated measures [59]. In MLM, treatment effects are examined as differences in trajectories of change between groups. A separate model will be estimated for each of the primary outcomes (dyadic adjustment, relationship satisfaction, and romantic attachment). Each model will include repeated measures of the outcome variable (level I) nested within individuals (level II), who are nested in couples (level III). The treatment effect will be examined at level III by including the treatment condition as a dichotomous predictor. Because the treatment effect is examined across multiple measurement points, a modified *d* (*d*_*GMA*_) [59] is used as a measure of the effect size_._ Using MLM has several advantages. It accounts for the non-independence in the repeated measures as well as between partners in the same couple. It also allows all available data to be included in the model, regardless of differences in assessment schedule or attrition. By including data from all participants, including those who discontinue voluntarily or who are removed by the research team, MLM analyses can be viewed as a form of intent-to-treat analysis.

To supplement our primary analyses, for each of our primary outcome variables, we will also use Jacobson and Truax’s method [60] to categorize each individual’s change as either clinically significant improvement, reliable improvement, reliable deterioration, or no reliable change. *Z*-tests will be used to compare the percent of individuals experiencing clinically significant change in the treatment and control conditions.

### Interim analyses {21b}

No interim analyses are planned.

### Methods for additional analyses (e.g., subgroup analyses) {20b}

The secondary objective of this study is to identify factors that influence the change trajectory or that act as potential mechanisms of change. To examine whether factors that are theorized to impact the change trajectory have an effect, the same multilevel model used to test the efficacy of treatment will be re-estimated with these factors included as predictors. To examine variables that may act as mechanisms of change, we will use multilevel mediation models using HLM with Bayesian estimation to examine the indirect effect of these variables (e.g., therapeutic alliance) on outcome.

### Methods in analysis to handle protocol non-adherence and any statistical methods to handle missing data {20c}

One of the primary benefits of using MLM is that it can easily accommodate differences in assessment schedules (non-adherence) or missing data. All available data from all participants is included in the analysis regardless of attrition status. If non-adherence to treatment protocol is identified as a problem, therapist treatment adherence scores (percent of interventions in the session that are identified as EFT interventions) will be entered as a level-III predictor. This will allow us to identify whether non-adherence alters the trajectory of change for the treatment group.

The main analysis will be run as an intention-to-treat analysis. However, in order to handle protocol non-adherence, per-protocol analyses will be also run, excluding couples that had fewer than ten sessions (i.e., less than half of the expected length of the treatment).

To address missing data from participants who drop out early, and other missing data, full information maximum likelihood (FIML) will be used in MLM models.

Clearly miskeyed or mistaken data entries will be interpreted and corrected if possible (i.e., an entry of “1q” on a questionnaire that only allows numeral entries from 1 to 4 may be interpreted as a “1” with an unintended “q” key stroke as well) and marked as missing if interpretation is not possible.

### Plans to give access to the full protocol, participant-level data, and statistical code {31c}

The full protocol is available at the webpage of the project [61]. Data and code will be available upon reasonable request once the corresponding results have been published.

## Oversight and monitoring

### Composition of the coordinating center and trial steering committee {5d}

The PI, the co-PI, and five members of the research team will compose the coordinating center. Among these five members, one will be in charge of the financial management and the recruitment process, another one will be in charge of the data quality and management, and the other three will be in supervising the day-to-day process, proving organizational support to therapists, supervisors, and couples. These last three, together with the PI, are the trial steering committee.

A member of the steering committee will be appointed as coordinator for each country, to offer direct support to therapists, supervisors, and couples throughout the trial. The coordinating center will have monthly or bimonthly meetings for quality control. The steering committee will have weekly or biweekly meetings.

All staff convened to the study, including PI and co-PI themselves and other therapists and supervisors, research team members, PhD students, and data analysts, will contribute to the smooth operation of the whole process. Funders are not implicated in the trial design and conduct, nor are they involved in the supervision.

### Composition of the data monitoring committee, its role, and reporting structure {21a}

A data monitoring committee is not included in the study protocol. The E(f)FECTS project is not a long-term trial as it has a clear intervention path, with a maximum of 21 couple therapy sessions. Thus, it has a clear predefined endpoint. Moreover, no adverse events or harm is expected from the couple therapy that will be provided. Participants with high levels of relationship distress are screened out of the study as are those with alcohol, substance, or significant mental illness.

### Adverse event reporting and harms {22}

See the section “Provisions for post-trial care {30}”.

### Frequency and plans for auditing trial conduct {23}

Oversight on protocol fidelity will be provided by the Universidad de Navarra Ethics Committee (UNAV-EC), which is an independent team, external to the research team or anyone from the staff involved in the study. This audit will take place at least annually.

### Plans for communicating important protocol amendments to relevant parties (e.g., trial participants, ethical committees) {25}

Any protocol amendments will be sent to the Ethics Committee (UNAV-EC) for reviews. Notification of any approved modifications will be forwarded to enrolled participants. The UNAV-EC will register any amendments and the steering committee will inform the enrolled couples.

## Dissemination plans {31a}

Results of this study will be published in scientific papers and presented in scientific international conferences. Moreover, we are going to present a summary of the findings in an E(f)FECTS project conference which would be recorded and shared open-access online through the webpage of the project (www.effects.es) or another open-access channel. Interviews in local newspapers in the countries involved in the study will be promoted, to disseminate the results to the public. Finally, a brief report with the main findings of the study will be shared with all the certified therapists or therapists in process of training in emotionally focused couple therapy, to improve their therapy implementation and the therapy outcomes.

## Discussion

Couple distress is common and is associated with decreased physical, mental, and emotional health as well as decreased job productivity [5]. While research has shown that couple therapy, broadly, and EFT in particular, are very effective in decreasing couple distress [10], this research has been conducted almost exclusively with English-speaking couples in the USA and Canada. Although Spanish is the second-most commonly spoken language in the world, there are no randomized controlled trials of couple therapy with this population. While professional organizations have called for culturally sensitive research and practice, the field has yet to answer these calls. The E(f)FECTS study aims to answer this call by understanding the efficacy of EFT in five Spanish-speaking countries. This study evaluates how emotionally focused couple therapy can improve couple adjustment, couple satisfaction, and secure attachment levels, as main outcomes, and considers many other dimensions as control, secondary outcomes or covariates.

As the first efficacy study of couple therapy in Spanish-speaking countries, this study is not without limitations. First, the comparison group is a no-treatment control. While answering the question about whether EFT is more effective than no treatment is an important step, the design does not test whether EFT is more effective than treatment as usual. This will be an important next step for the field to take. Second, the country subgroup sample size will not be large enough to make confident comparisons of the efficacy of treatment across sites. Finally, as all therapists have spent significant amounts of time and money to become certified EFT therapists, there may be an allegiance effect that is not controlled for in this study.

Despite these limitations, as the first controlled trial of couple therapy in the Spanish-speaking population, the results of this study will be a milestone in the treatment of couple distress in Argentina, Costa Rica, Guatemala, Mexico, and Spain. An additional contribution of the E(f)FECTS project is that the therapists delivering the intervention have been trained by different trainers and supervisors and have different degrees of experience. Previous trials of EFT have been carried out with a very small group of therapists, often students with advanced training or by experts in the model, limiting the generalizability of results. In addition to the primary outcome variables, this project will also examine a number of secondary constructs allowing us to understand the impact of couple treatment on a wider range of outcomes. Most of these variables are measured repeatedly throughout the study, allowing us to better understand not just whether treatment works, but what the change process looks like. The repeated assessment coupled with video recordings of the therapy will provide a rich understanding of this change and allow us to examine how EFT is being practiced in culturally competent ways by therapists in their local communities. These advances will allow the E(f)FECTS project to contribute in significant ways to the field of couple therapy, broadening access to evidence-based couple therapy in the Spanish-speaking cultural context.

## Trial status

Trial registration: NCT04277325; February 20, 2020.

Participant recruitment began in September 2021 (for Spain), February 2022 (for Costa Rica and Mexico), and March 2022 (for Argentina and Costa Rica). At the time of this manuscript submission, recruitment is actively occurring in all countries except Spain. The intervention will end around 6 months after the start date in each country. Follow-up for the intervention group will end 2 years after the end of the intervention.

## Supplementary Information

**Additional file 1: Supplementary Table 1.** WHO trial registration dataset.

## Data Availability

All data requests should be submitted to the corresponding author for consideration. Access to anonymized data may be granted following review.

## Abbreviations

IAS-C4: Intersession Alliance Scale-Couple
ABAQ: Attachment Based Alliance Questionnaire
AP: Authoritative Parenting subscale of the Parenting Style and Dimensions Questionnaire Short form (PSDQ)
APA: American Psychological Association
BARE: Brief Accessibility, Responsiveness, and Engagement Scale
BYU: Brigham Young University
CONSORT: Consolidated Standards of Reporting Trials
CORE: Clinical Outcomes in Routine Evaluation
CSI: Couple Satisfaction Inventory
DAS: Dyadic Adjustment Scale
DASS: Depression Anxiety Stress Scale
ECR: Experience in Close Relationships Inventory
EFT: Emotionally Focused Therapy for couples
FIML: Full information maximum likelihood
GLMs: General linear models
HMT: Hold Me Tight
LGC: Latent growth curve
MANCOVA: Multivariate analysis of covariance
MLM: Multilevel modeling
PHQ: Patient Health Questionnaire
PI: Principal investigator
PROMIS: Patient-Reported Outcomes Measurement Information System
PSRQ: Post Session Resolution Questionnaire
PTSD: Post-traumatic stress disorder
RCI: Reliable Change Index
RELATE: RELATionship Evaluation Questionnaire
RFQ: Reflective Functioning Questionnaire
SD: Sexual Dissatisfaction subscale of Marital Satisfaction Inventory (MSI)
S-DSI: Spanish Differentiation of Self Inventory
SEM: Structural equation modeling
SFTP: Secure file transfer protocol
SLEs: Stressful life events
UCLA LS-R: Revised UCLA Loneliness Scale
UNAV: University of Navarra
UO: University of Ottawa
UQO: University of Québec au Outaouais
WAI-Co-SF: Working Alliance Inventory for Couples Short Form
