# Supplementary material for: Efficacy of Emotionally Focused Therapy among Spanish-speaking couples: study protocol of a randomized clinical trial in Argentina, Costa Rica, Guatemala, Mexico, and Spain
Source: Trials. 2022 Oct 22;23:891. doi: 10.1186/s13063-022-06831-7 (PMC9587591; doi:10.1186/s13063-022-06831-7)
Supplement: Supplementary file 1 — Additional file 1: Supplementary Table 1. WHO trial registration dataset. [file 13063_2022_6831_MOESM1_ESM.docx]

| **Supplementary Table 1** WHO trial registration dataset | |
| --- | --- |
| 1. Primary registry and trial identifying number | ClinicalTrials.gov: NCT04277325 |
| 1. Date of registration in primary registry | February 20, 2020 |
| 1. Secondary identifying numbers | UNAV.2019.149  EASH-ICS.2020.1 |
| 1. Source(s) of Monetary or Material Support | Universidad de Navarra (UNAV), Spain  Brigham Young University (BYU), USA  International Centre for Excellence in Emotionally Focused Therapy (ICEEFT), Ottawa, Canada |
| 1. Primary sponsor | Universidad de Navarra, Spain |
| 1. Secondary sponsor(s) | Brigham Young University, USA |
| 1. Contact for public queries | Dr. Martiño Rodríguez-González (effects@unav.es) |
| 1. Contact for Scientific Queries | Dr. Martiño Rodríguez-González (effects@unav.es) |
| 1. Public title | Efficacy of Emotionally Focused Therapy Among Spanish Speaking Couples (E(f)FECTS) |
| 1. Scientific title | Efficacy of Emotionally Focused Therapy Among Spanish Speaking Couples (E(f)FECTS) |
| 1. Countries of recruitment | Argentina, Costa Rica, Guatemala, México, and Spain |
| 1. Health Condition(s) or Problem(s) Studied | Couple relationship distress, insecure couple attachment |
| 1. Intervention(s) | Treatment group: 19-21 sessions of Emotionally Focused Therapy (EFT)  Control group: no treatment |
| 1. Key inclusion-exclusion criteria | **Inclusion Criteria**   1. Couples who have been in an exclusive relationship and living together for at least 1 year 2. Both members of the couple must be over 25 years old (there is no upper limit for participants’ age) 3. Both members are willing to participate in all aspects of the study, including completing questionnaires, being videotaped in therapy, attending therapy, participating in the follow-up after treatment has been completed 4. Both members of the couple are native Spanish speakers and have lived in one of the included countries (Argentina, Costa Rica, Guatemala, Mexico, or Spain) for a minimum of 5 years prior to participating 5. The average score of the couple’s dyadic adjustment, measured by the Dyadic Adjustment Scale (DAS, see Outcomes section below) falls between 80 and 100 (from mildly to moderately distressed).   **Exclusion Criteria**  Either partner:   1. is receiving current treatment through psychotherapy at the time of recruitment or anticipates doing so outside of the proposed study within the next six months 2. has been previously diagnosed with any psychotic, somatoform, or dissociative disorder 3. is taking medication known to treat psychosis, somatoform, psychotic or dissociative disorders or is taking a psychotropic medication 4. is misusing drugs or alcohol, defined as frequent (more than once a week) and maintained (for more than a year) use that has led to a work or personal problem. 5. has a diagnosis of a neurodevelopmental, neurocognitive, personality, or paraphilic disorder 6. reports having been arrested or in prison in the past 3 months 7. reports losing her/his employment due to alcohol or drug related problems 8. reports an episode of sexual assault (as victim or perpetrator) in their life during the last 2 years 9. reports current physical or sexual violence in their relationship 10. is currently involved in an affair which she/he is unwilling to disclose to her/his partner and/or terminate 11. has or anticipates circumstances which will make attending therapy sessions unlikely, such as major surgery expected in the next 3 months, or moving to a new area in the near future, etc. 12. is a psychotherapist in active clinical practice 13. has a direct knowledge of EFT because they are currently receiving training or have been trained in EFT |
| 1. Study Type | Study Type: Interventional (Clinical Trial)  Allocation: Randomized  Masking: None (Open Label)  Assignment: Parallel  Purpose: Treatment |
| 1. Date of first enrolment | September 2021 |
| 1. Sample size | 136 participants |
| 1. Recruitment status | Complete (July, 2022) |
| 1. Primary Outcome(s) | Outcome Name: Dyadic adjustment  Metric/method of measurement: Dyadic Adjustment Scale (DAS-32 and DAS-4)  Timepoints: See Table 2  Outcome Name: Couple Satisfaction  Metric/method of measurement: Couple Satisfaction Inventory (CSI-16)  Timepoints: See Table 2  Outcome Name: Romantic attachment  Metric/method of measurement: Experiences in Close Relationships Questionnaire (ECR-36)  Timepoints: See Table 2  (See Outcomes {12} section). |
| 1. Key Secondary Outcomes | - General Health Questions (Health-4) - Patient Health Questionnaire (PHQ-15) - Depression Anxiety Stress Scale (DASS-21) - Sexual Dissatisfaction subscale of the Marital Satisfaction Inventory (SD-13) - UCLA Loneliness Scale Revised - Short version (UCLA LS-R-8) - Reflective Functioning Questionnaire (RFQ-8) - Authoritative Parenting subscale (RELATE)-(AP-15) - Sleep Quality (Sleep-8) - CORE Outcome Measure short form (CORE-10) - NEO Five Factor Inventory (NEO-N12) - Spanish Differentiation of Self Inventory (S-DSI-26) - RELATE measure (RELATES-55) - Stressful Life Events (SLEs-15) - The Brief Accessibility, Responsiveness, and Engagement Scale (BARE-12)   (See Outcomes {12} section). |
| 1. Ethics Review | Status: Approved  Date of approval: October 15, 2019  Name and contact details of ethics committees:  Research Ethics Committee of the Universidad de Navarra  Av. Pío XII, 36  31008 Pamplona (Spain)  Phone:+34 948255400  Email: ceic@unav.es |
| 1. Completion Date | January 2023 (Estimated) |
| 1. Summary results | No results yet |
| 1. IPD sharing statement | Plan to share Individual participant data (IPD): No. |
